# Factors associated with insufficient response to acute treatment of migraine in Japan: analysis of real-world data from the Adelphi Migraine Disease Specific Programme

**DOI:** 10.1186/s12883-020-01848-4

**Published:** 2020-07-08

**Authors:** Koichi Hirata, Kaname Ueda, Wenyu Ye, Yongin Kim, Mika Komori, James Jackson, Sarah Cotton, Narayan Rajan, Tamas Treuer

**Affiliations:** 1 Department of Neurology, Dokkyo Medical University, Tochigi, Japan; 2 Eli Lilly Japan K.K, Kobe, Japan; 3 Eli Lilly and Company, Indianapolis, IN USA; 4 Adelphi Real World, Adelphi Mill, Bollington, UK; 5 Eli Lilly Australia, West Ryde, NSW Australia; 6 Lilly Hungária Kft, Budapest, Hungary

**Keywords:** Adelphi Migraine Disease Specific Programme, Japan, Migraine, Quality of life, Retrospective study, Treatment satisfaction, Work productivity

## Abstract

**Background:**

Real-world data on sufficient/insufficient response, and predictors of insufficient response, to acute treatments for migraine are limited in Japan. This study aimed to identify factors associated with insufficient response to acute treatment of migraine by exploring significant differences between people with migraine who sufficiently/insufficiently respond to prescribed acute treatment in Japan.

**Methods:**

This was a retrospective analysis of 2014 Adelphi Migraine Disease Specific Programme cross-sectional survey data collected from physicians and their consulting adult patients with migraine in Japan. Insufficient responders to prescribed acute treatment were patients who achieved headache pain freedom within 2 h of acute treatment in no more than three of their last five migraine attacks. Factors associated with insufficient response to prescribed acute migraine treatment were identified using backward logistic regression.

**Results:**

Overall, 227/538 (42.2%) patients were classified as insufficient responders to prescribed acute migraine treatment. Significantly more insufficient responders than sufficient responders had consulted a neurologist or a migraine/headache specialist, and had chronic migraine or medication-overuse or tension-type headaches (*p* < 0.05). More insufficient responders than sufficient responders reported taking acute treatment when/after the pain started (77.0 vs. 68.9%) than at first sign of migraine (*p* < 0.05). Compared with sufficient responders, insufficient responders reported a significantly higher mean ± standard deviation (SD) Migraine Disability Assessment total score (12.7 ± 23.3 vs. 5.8 ± 10.4, *p* < 0.001) and lower quality of life (EuroQol-5 Dimensions utility score 0.847 ± 0.19 vs. 0.883 ± 0.16, *p* = 0.024). Factors significantly associated with insufficient response to acute treatment included seeing a neurologist versus an internist (odds ratio [OR] 1.93; 95% confidence interval [CI] 1.29–2.88; *p* = 0.002), taking acute medication when/after pain started versus at first sign of migraine (OR 1.65; 95% CI 1.05–2.60; *p* = 0.030), a higher MIDAS total score (OR 1.04; 95% CI 1.02–1.06; *p* < 0.001), and presence of comorbid cardiovascular disease (OR 0.53; 95% CI 0.28–0.98; *p* = 0.044).

**Conclusions:**

Many people with migraine in Japan struggle to adequately treat migraine attacks with prescribed acute medication and exhibit high levels of unmet need for acute treatment. Optimized management strategies utilizing existing therapeutic options as well as additional effective therapeutic options for migraine are required to improve symptoms and quality of life.

## Background

Migraine is highly prevalent worldwide, with an average global prevalence at the community level of 11.6% (10.1% in Asia) [1]. In 2016, migraine was reported to be the second leading cause of years lived with disability (YLD) globally and the fourth leading cause of YLD in Japan [2].

Management options include acute treatment to relieve pain during an attack or to limit an attack, emergency treatment, and preventive medication [3]. According to current International Headache Society (IHS) guidelines, the 2-h pain-free response provides the most clinically relevant information about the efficacy of an acute treatment for migraine [3]. In Japan, medications recommended by the Japanese Society of Neurology (JSN) and the Japanese Headache Society (JHS) for the acute treatment of a migraine attack include acetaminophen, nonsteroidal anti-inflammatory drugs (NSAIDs), triptans, ergotamines, and antiemetics [4].

There is evidence that current treatment approaches are not sufficiently meeting the needs of people with migraine. In a US longitudinal population-based study (American Migraine Prevalence and Prevention [AMPP]), > 40% of people with episodic migraine were found to have at least one unmet need with their current acute treatment, which included the domain satisfaction with therapy (assessed as lack of efficacy, tolerance, or overall satisfaction with the medication) [5]. In that study, 56% of individuals with episodic migraine reported an insufficient treatment response to acute medication (insufficient 2-h pain-free response) [6]. It should be noted that individuals with episodic migraine who do not respond to acute treatments are at risk of progressing to chronic migraine [7].

Real-world data on sufficient/insufficient response and predictors of insufficient response to acute treatments for migraine in individuals in Japan are limited. The 2014 Japan Adelphi Migraine Disease Specific Programme (DSP) sought to provide a real-world understanding of information on the clinical characteristics, disease burden, and treatment patterns of patients with migraine being treated in clinical practice in Japan. In a recently published first retrospective analysis of Japan Adelphi Migraine DSP data [8], patients being treated for episodic or chronic migraine were found to have unmet needs with current acute and preventive therapy in line with the above-mentioned US survey findings.

The aims of the current retrospective analysis using the Japan Adelphi Migraine DSP data were to identify any significant differences in demographics, clinical characteristics, disease burden, and treatment patterns between patients with migraine who sufficiently/insufficiently responded to acute treatment and to explore factors associated with insufficient response to acute treatment of migraine in Japan.

## Methods

### Derivation of data

This research was a retrospective analysis of cross-sectional survey data on migraine treatment practice, patient demographics, clinical features and outcomes, healthcare utilization, work productivity, and health-related quality of life (HRQoL) collected as part of the Japan Adelphi Migraine DSP in 2014. Data were collected cross-sectionally using standardized DSP methodology over the period January to March 2014. Full details of the methodology of DSPs and the primary analysis of DSP data have been reported [8, 9].

Participating physicians were required to recruit ten patients who had a diagnosis of migraine. The first nine patients were to be consecutive, but to achieve a 10% oversampling of patients who had failed at least one prior preventive treatment, the tenth patient had to meet this requirement and may not have been consecutive.

The population surveyed in the Japan Adelphi Migraine DSP included both physicians (internists and neurologists; either group could include migraine/headache specialists) treating people with migraine and their migraine-diagnosed adult patients who were actively seeking care from their healthcare provider.

For each patient, physicians were required to record detailed information on patient demographics, headache diagnoses (e.g., medication-overuse, tension-type, chronic, episodic), comorbidities, clinical features, and treatment of migraine using a patient record form (PRF). Data from PRFs are referred to as physician-reported data throughout. The physician invited each patient to complete a confidential patient self-completion (PSC) form, which asked questions about the patient’s demographics, and patient-reported outcomes such as headache-related disability over the past 3 months (using the Migraine Disability Assessment [MIDAS] test [10, 11]), HRQoL (using the EuroQol-5 Dimensions [EQ-5D] questionnaire [12]), and work productivity and usual activity impairment due to migraine (using the Work Productivity and Activity Impairment [WPAI]: Migraine V2.0 questionnaire [13]), translated into Japanese by an accredited translation agency) (data from PSC forms are referred to as patient-reported data throughout). The PSC was also used by patients to record their response to prescribed acute treatment (assessed as the achievement of headache pain freedom within 2 h of acute treatment) in their last five migraine attacks. The question asked was, “In approximately how many migraine attacks would you say your prescription acute medicine stops the migraine pain entirely within 2 hours of taking the medication?” Patients could select from 0, 1, 2, 3, 4, or 5 out of every five attacks in response.

### Patient cohorts

Two cohorts (sufficient responders/insufficient responders) were defined on the basis of their response to prescribed acute treatment in their last five migraine attacks. Sufficient responders were defined as those who achieved headache pain freedom within 2 h of acute treatment (a recognized efficacy endpoint in clinical trials assessing acute treatments for migraine [14]) in at least four of their last five migraine attacks. Insufficient responders were defined as patients who achieved headache pain freedom within 2 h of acute treatment in no more than three of their last five migraine attacks.

### Statistical methods

Deidentified and quality-checked data from the 2014 Japan Adelphi Migraine DSP for all patients meeting the eligibility criteria for the study (adults diagnosed with migraine who were actively seeking care from their healthcare provider) were included in analyses. Because these were retrospective analyses on available data from the 2014 Japan Adelphi Migraine DSP, no statistical power calculation was conducted prior to the study.

Demographics, clinical characteristics, disease burden, and treatment patterns among sufficient and insufficient responders to prescribed acute treatment were summarized using descriptive statistics. Continuous measures (presented as means with standard deviations [SDs]) were assessed using two sample t-tests; categorical measures (presented as numbers and proportions) were assessed using Fisher’s exact (for small sample sizes) or chi-squared tests.

Backward logistic regression was used to identify factors associated with insufficient response versus sufficient response. The variables we considered for inclusion in the backward logistic regression were either based on statistical significance from bivariate tests or measures that may be relevant to a person’s experience with migraine attacks. Candidate predictor variables included age, sex, type of physician, employment status, time to migraine diagnosis (in weeks), living status, smoking status, comorbidities (depression, anxiety, cardiovascular disease), insurance plan, currently prescribed acute medication, time of administration of prescribed acute treatment, migraine with aura, change in migraine headache days per month before prescribed acute treatment, migraine headache days per month, patient level of impairment in the last 6 months, number of prescribed acute medications, number of prescribed preventive therapies, and MIDAS total score. In backward selection, a significance level of 0.1 was required for a variable to be retained in the model. Adjusted odds ratios (ORs), 95% confidence intervals of ORs (CIs), *p*-values, and c-statistic from the final model are reported.

Summary statistical information was based on non-missing data. Statistical tests were conducted at a two-sided 5% significance level. Analyses were conducted using SAS version 9.3 (SAS Institute, Cary, NC, USA).

## Results

**Fig. 1:**
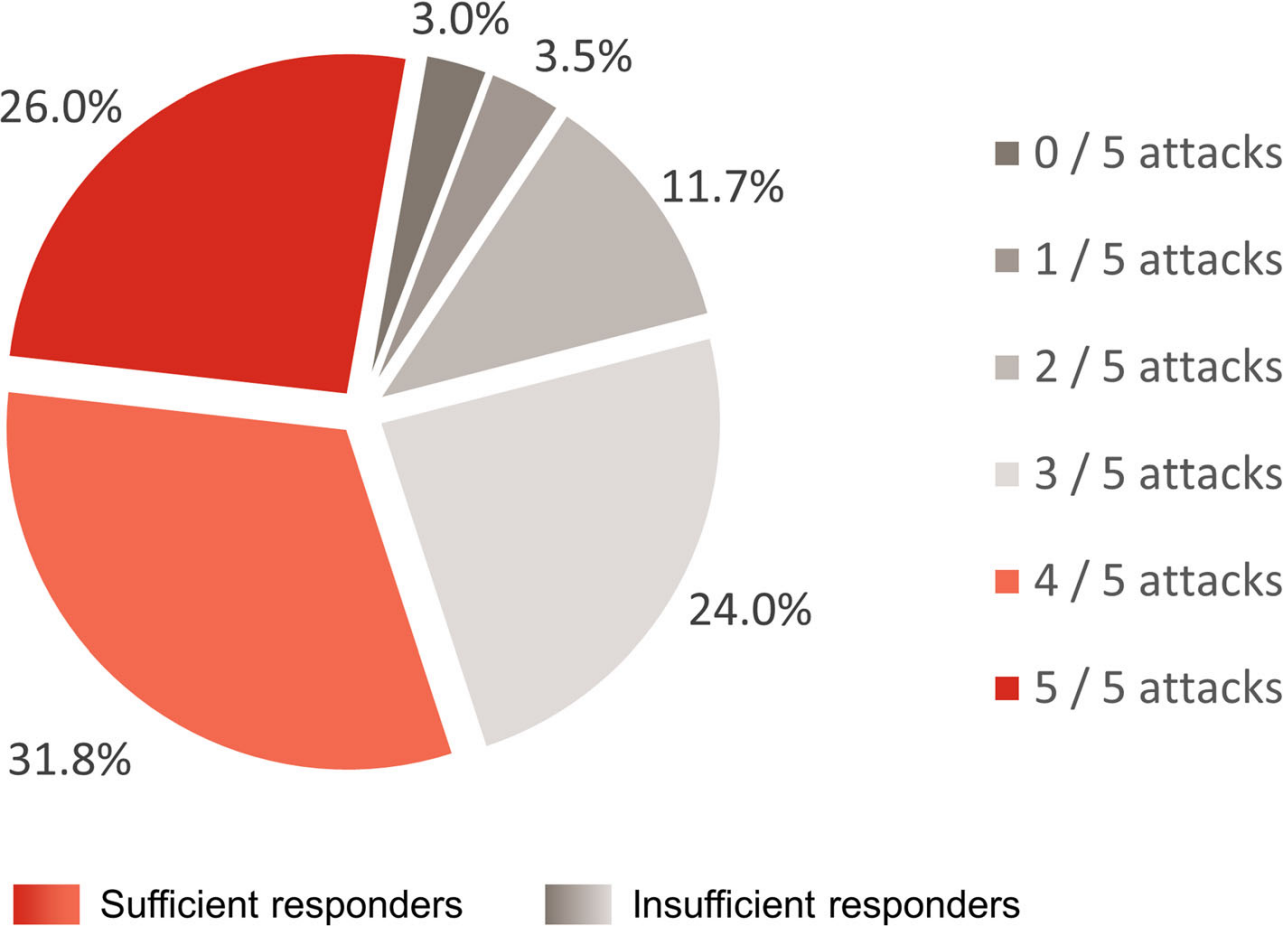
Distribution of sufficient responders and insufficient responders to acute treatment for migraine: patient-reported data. Sufficient responders were defined as those who achieved headache pain freedom within 2 h of acute treatment in at least four of their last five migraine attacks; insufficient responders were defined as those who achieved headache pain freedom within 2 h of acute treatment in no more than three of their last five migraine attacks

Of 538 patients who provided information on their response to prescribed acute treatment for their last five migraine attacks, 227 were classified as insufficient responders (42.2%) and 311 as sufficient responders (57.8%) (Fig. 1). Age and sex were well balanced between insufficient responder and sufficient responder groups. Insufficient responders and sufficient responders also demonstrated a similar distribution in the number of migraine headache days/month experienced (0–3, 4–7, or ≥ 8). Similar proportions of insufficient responders and sufficient responders were reported by physicians as having at least one comorbidity, the most common being hypertension and hyperlipidemia. There was no significant difference in the frequency of any comorbidity between insufficient responders and sufficient responders (Table 1).

**Table 1:** Patient characteristics

	Insufficient responders (*N* = 227)	Sufficient responders (*N* = 311)	Total (*N* = 538)
Age (years, mean ± SD)	43.6 ± 13.4	44.5 ± 14.1	44.1 ± 13.8
Female, n (%)	180 (79.3)	235 (75.6)	415 (77.1)
Smoking status: current or prior smoker, n (%)	45 (20.3)	71 (23.9)	116 (22.4)
Neurologist consultation, n (%)*	**117 (51.5)**	**98 (31.5)**	**215 (40.0)**
**Migraine/headache specialist**^**a**^, n (%)*	**103 (45.4)**	**101 (32.5)**	**204 (37.9)**
Employed, n (%)	138 (63.0)	198 (64.5)	336 (63.9)
Number of migraine headache days/month, n (%)			
0–3	103 (59.9)	151 (63.7)	254 (62.1)
4–7	44 (25.6)	58 (24.5)	102 (24.9)
≥ 8	25 (14.5)	28 (11.8)	53 (13.0)
Chronic/episodic migraine, n (%)*			
Chronic migraine	20 (8.8)	11 (3.6)	31 (5.9)
Episodic migraine	206 (91.2)	291 (96.4)	497 (94.1)
Migraine with aura, n (%)	82 (36.1)	121 (38.9)	203 (37.7)
Family history of migraine: parent, n (%)	45 (20.5)	55 (17.8)	100 (18.9)
Rebound headache or medication-overuse headache, n (%)*	**15 (6.6)**	**9 (2.9)**	**24 (4.5)**
Tension-type headache, n (%) *	**63 (27.8)**	**51 (16.4)**	**114 (21.2)**
Comorbidity (yes), n (%)	103 (45.4)	149 (47.9)	252 (46.8)
Hypertension^b^	23 (22.3)	46 (30.9)	69 (27.4)
Hyperlipidemia^b^	20 (19.4)	32 (21.5)	52 (20.6)
Anxiety^b^	18 (17.5)	23 (15.4)	41 (16.3)
Sleep disorders^b^	15 (14.6)	21 (14.1)	36 (14.3)
Asthma/COPD/allergic rhinitis^b^	15 (14.6)	17 (11.4)	32 (12.7)
Depression^b^	13 (12.6)	11 (7.4)	24 (9.5)
GI problems/dyspepsia^b^	10 (9.7)	19 (12.8)	29 (11.5)
Other^b^	16 (15.5)	22 (14.8)	38 (15.1)
Cardiovascular disease,^b,c^	23 (22.3)	47 (31.5)	70 (27.8)

Physician-reported data

*p < 0.05 between insufficient responders and sufficient responders (variables with significant differences in bold). For categorical measures, chi-squared or Fisher’s exact test was used. For continuous measures, t-test was used

^a^Patients visited migraine/headache specialists who were either neurologists (*n* = 164; 30.5% of patients) or internists (*n* = 40; 7.4% of patients)

^b^Percentages calculated based on the respective number of patients in each group with any comorbidity. Percentages are calculated as proportion of non-missing data. Comorbidities listed are those occurring in ≥10% of patients experiencing a comorbidity. Chronic migraine was defined as (≥15 headache days per month [15]); episodic migraine was defined as not fulfilling chronic migraine criteria [16]

^c^Cardiovascular disease is a derived variable that includes angina (seen in 1/2 insufficient/sufficient responders, respectively), hypertension (data in table), ischemic heart disease (seen in 0/2 insufficient/sufficient responders, respectively), post myocardial infarction (no occurrences in either group, and congestive heart failure (no occurrences in either group)

*COPD* chronic obstructive pulmonary disease; *GI* gastrointestinal; *SD* standard deviation

Information on migraine-related symptoms, including the site and severity of pain, is given in Supplementary Table 1. Among patients reporting migraine-related symptoms, the distribution of severity (ranging from none to severe) differed significantly (*p* < 0.05) in insufficient responders versus sufficient responders for unilateral pain, bilateral pain, pain worsened by activity, sensitivity to smell, sensory aura, visual aura, speech disturbance, muscle weakness/fatigue, and light headedness (Supplementary Table 1).

Significant (*p* < 0.05) differences between the characteristics of the two groups included more insufficient responders than sufficient responders having consulted a neurologist or a migraine/headache specialist and having a clinical diagnosis of chronic migraine or exhibiting medication-overuse or tension-type headaches (physician-reported data) (Table 1).

The distribution of number of prescribed acute treatment regimens (0, 1, 2, or ≥ 3) did not differ significantly between insufficient responders and sufficient responders (Table 2). Current use of NSAIDs (including in combinations) and triptans as prescribed acute medication was also similar between insufficient responders and sufficient responders. No patient was using an opioid as prescribed acute medication. However, use of over-the-counter and/or prescribed acute medication differed significantly between groups (*p* < 0.05; Table 2).

**Table 2:** Physician-reported current acute treatment patterns in insufficient responders/sufficient responders to acute treatment for migraine

	Insufficient responders (*N* = 227)	Sufficient responders (*N* = 311)	Total (*N* = 538)
Number of prescribed acute regimens ever (% of total acute prescriptions)			
0	4 (1.8)	1 (0.3)	5 (0.9)
1	147 (64.8)	211 (68.1)	358 (66.7)
2	50 (22.0)	74 (23.9)	124 (23.1)
≥ 3	26 (11.5)	24 (7.7)	50 (9.3)
Prescribed acute medication type, n (%)^a^			
NSAIDs (including in combinations)	99 (43.6)	115 (37.0)	214 (39.8)
Triptans	171 (75.3)	214 (68.8)	385 (71.6)
Opioid analgesics (including in combinations)	0	0	0
Patient currently taking OTC medications, n (%)	12 (6.5)	22 (9.0)	34 (7.9)
Currently taking OTC and/or prescribed acute*			
Prescribed acute treatment only	171 (91.9)	220 (89.8)	391 (90.7)
OTC and prescribed acute	11 (5.9)	22 (9.0)	33 (7.7)
OTC only	1 (0.5)	0	1 (0.2)
Taking neither	3 (1.6)	3 (1.2)	6 (1.4)

Reported data are number and percentage of patients, with percentages calculated as proportion of non-missing data

*p < 0.05 between insufficient responders and sufficient responders. Chi-squared or Fisher’s exact test was used

^a^Occurring in ≥5% of patients

*NSAID* nonsteroidal anti-inflammatory drug; *OTC* over the counter

Patient-reported timing of administration of acute therapy differed significantly (*p* < 0.05) between the two groups, with more insufficient responders than sufficient responders taking their acute treatment when/after the pain started (77.0% vs. 68.9%) rather than at first sign of a migraine (Table 3). Insufficient responders and sufficient responders answered significantly differently (*p* < 0.05) when asked whether they would like to continue using their currently prescribed acute medication (e.g., “definitely yes”: 21.6% vs. 43.1%) (Table 3). Insufficient responders were also more likely than sufficient responders to need to take extra doses of their prescribed acute medication to relieve pain symptoms or symptoms of a migraine attack (41.1% vs. 18.7%, *p* < 0.05) (Table 3). Among patients who needed to take extra doses of acute medication, the number of times extra doses were taken for the last ten migraine attacks was significantly higher in insufficient responders than in sufficient responders (3.34 vs. 2.14, p < 0.05) (Table 3).

**Table 3:** Patient-reported usage of current acute treatment in insufficient responders/sufficient responders to acute treatment for migraine

	Insufficient responders (*N* = 227)	Sufficient responders (*N* = 311)	Total (*N* = 538)
Time of administration of acute therapy, n (%)*			
At first sign of a migraine	49 (23.0)	94 (31.1)	143 (27.8)
When/after the pain starts	164 (77.0)	208 (68.9)	372 (72.2)
Continue using currently prescribed acute medication, n (%)*			
Definitely yes	48 (21.6)	132 (43.1)	180 (34.1)
Probably yes	121 (54.5)	147 (48.0)	268 (50.8)
Do not know	43 (19.4)	24 (7.8)	67 (12.7)
Probably not	10 (4.5)	2 (0.7)	12 (2.3)
Definitely not	0	1 (0.3)	1 (0.2)
Ever needs to take extra doses to relieve pain/migraine symptoms, n (%)*	85 (41.1)	52 (18.7)	137 (28.2)
Number of times extra doses of a prescribed acute medication were taken for the last ten migraine attacks, mean ± SD*^a^	3.34 ± 2.1	2.14 ± 1.9	2.89 ± 2.1

Reported data are number and percentage of patients, unless stated otherwise, with percentages calculated as proportion of non-missing data

^*^p < 0.05 between insufficient responders and sufficient responders. For categorical measures, chi-squared or Fisher’s exact test was used. For continuous measures, t-test was used

^a^Assessed in patients needing to take extra doses to control pain/migraine symptoms

*SD* standard deviation

**Fig. 2:**
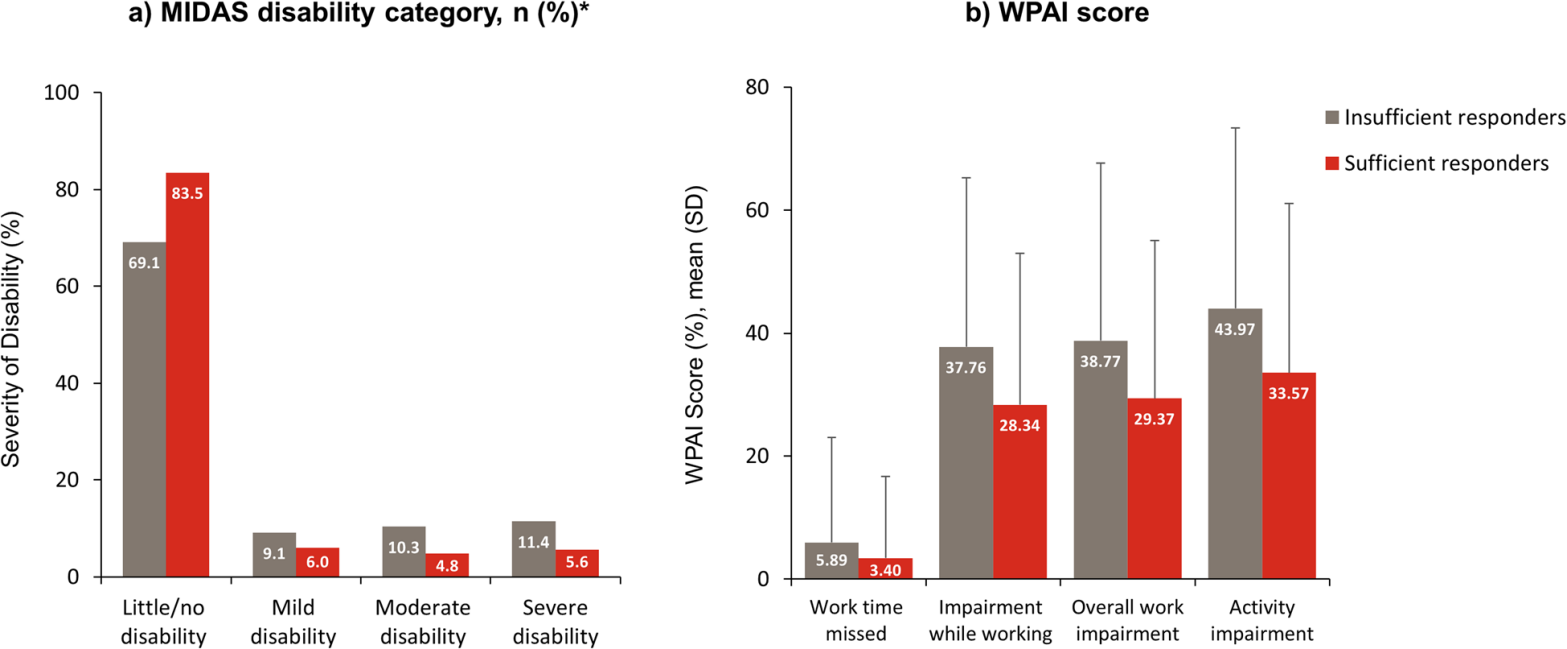
Patient-reported outcomes in insufficient responders and sufficient responders to acute treatment for migraine. *p < 0.05 between insufficient responders and sufficient responders. For categorical measures, chi-squared or Fisher’s exact test was used. For continuous measures, t-test was used. Percentages are calculated as proportion of non-missing data. *MIDAS* Migraine Disability Assessment; *WPAI* Work Productivity and Activity Impairment

Compared with sufficient responders, insufficient responders to acute treatment reported significantly worse disability, as indicated by a higher mean ± SD MIDAS total score (12.7 ± 23.3 vs. 5.8 ± 10.4, *p* < 0.001), and quality of life, as indicated by lower mean ± SD EQ-5D visual analog (67.6 ± 17.7 vs. 75.5 ± 17.2, p < 0.001) and utility (0.847 ± 0.19 vs. 0.883 ± 0.16, *p* = 0.024) scores. In addition, headache-related disability (Fig. 2a) and the impact of headache on impairment at work, overall work impairment, and activity impairment, but not work time missed (WPAI scores) (Fig. 2b), were significantly greater among insufficient responders than sufficient responders (*p* < 0.05 for all comparisons).

Physicians considered that a significantly smaller proportion of insufficient responders than sufficient responders experienced much improvement in their level of impairment over the past 6 months (26.7% vs. 35.5%, p < 0.05) and impairment worsened slightly in significantly more insufficient responders than sufficient responders over the same period (5.3% vs. 2.0%, respectively, p < 0.05) (Supplementary Figure 1).

**Fig. 3:**
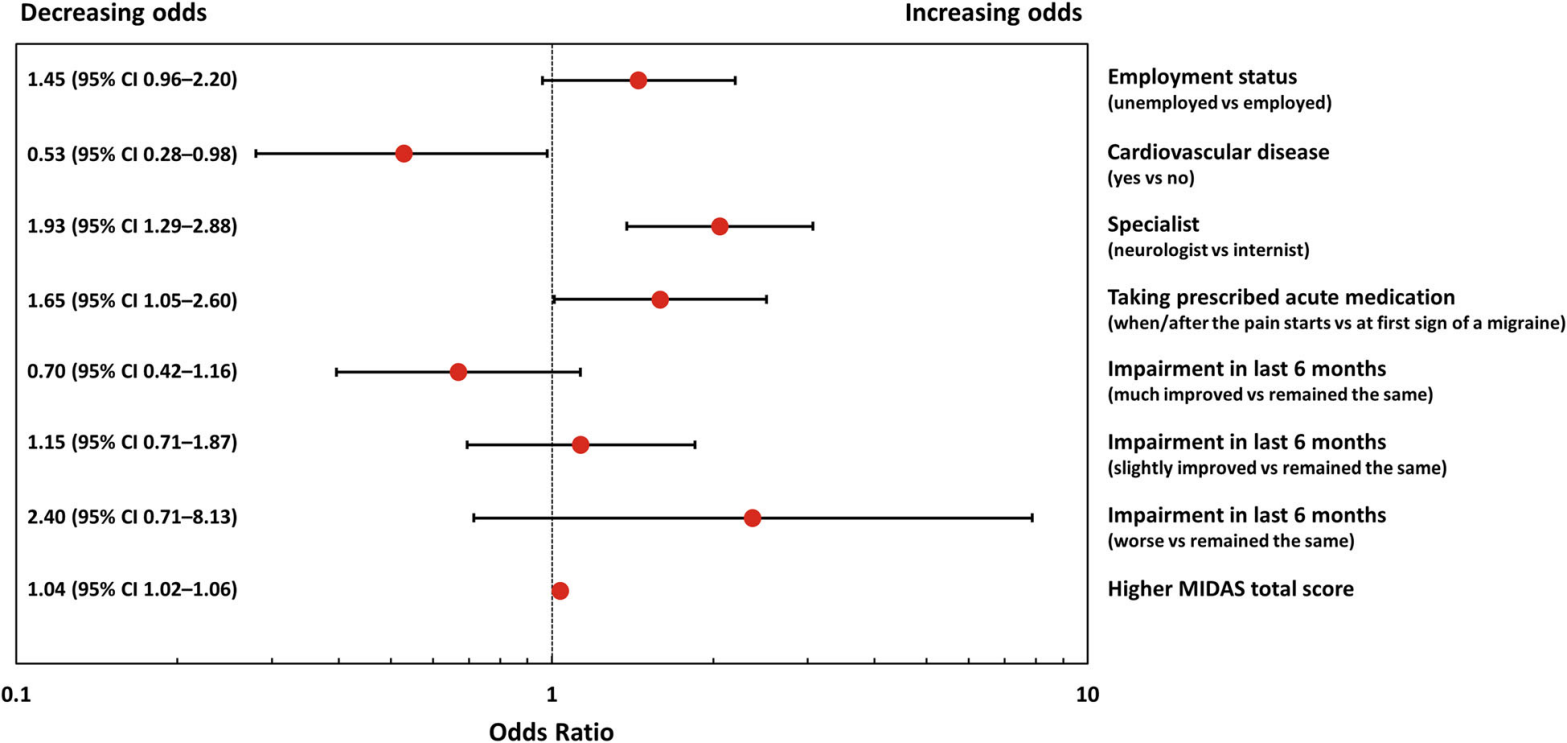
Factors associated with insufficient response in backward logistic regression analysis. Variables included in the logistic model but not found to be significant included age, sex, employment status, time to migraine diagnosis (in weeks), living status, smoking status, comorbidities (depression, anxiety, cardiovascular disease), insurance plan, currently prescribed acute medication, migraine with aura, change in migraine headache days per month before prescribed acute treatment, migraine headache days per month, number of prescribed acute medications, and number of prescribed prophylaxis therapies. Odds ratio > 1 with 95% CI not crossing 1 indicates a significant factor associated with an insufficient response to acute treatment; odds ratio < 1 with 95% CI not crossing 1 indicates a significant factor for a response to acute treatment. Cardiovascular disease includes angina, hypertension, ischemic heart disease, post myocardial infarction, and congestive heart failure. *CI* confidence interval; *MIDAS* Migraine Disability Assessment

Factors significantly (p < 0.05) associated with insufficient response to prescribed acute treatment using backward logistic regression are shown in Fig. 3. The odds of being an insufficient responder to acute treatment was higher for patients who consulted a neurologist than for patients who consulted an internist (OR 1.93; 95% CI 1.29–2.88; *p* = 0.002). Patients who took acute prescribed medication when/after pain started also had higher odds of being insufficient responders than those who took acute prescribed medication at first sign of migraine (OR 1.65; 95% CI 1.05–2.60; *p* = 0.030). Patients with cardiovascular disease had lower odds of being insufficient responders than those who reported no cardiovascular disease (OR 0.53; 95% CI 0.28–0.98; *p* = 0.044). Odds of being an insufficient responder increased 4% for every one-unit increase in total MIDAS score (OR 1.04; 95% CI 1.02–1.06; *p* < 0.001) (Fig. 3). The c-statistic from the final model was 0.7, indicating that the goodness of fit of the model was high.

## Discussion

The demographic profile of this real-world population with migraine (mean age 44 years; ~ 75% female) was generally representative of the global migraine population [17, 18]. In this real-world study, over 40% of patients were classified as insufficient responders to prescribed acute treatment (based on patient self-reported achievement of headache pain freedom within 2 h post first-dose in up to three of their last five migraine attacks).

Our findings support those of other real-world studies, including Adelphi Migraine DSPs conducted using the same methodology as that used in the current study. A 2014 US Adelphi Migraine DSP found that insufficient responders to acute therapy comprised 34% of the study population [19], and the same percentage of insufficient responders to acute treatment with triptans (34%) was found in a recent analysis of data from a 2017 Adelphi Migraine DSP conducted in the USA, France, Germany, Italy, Spain, and the UK [20].

The slightly higher proportion (56%) of insufficient responders to acute treatment reported by the US AMPP longitudinal population-based study [6] is possibly due to the use of a different definition of insufficient response in that survey: pain-free response achieved < 50% of the time. Clinical trial and observational study data have consistently reported that 30–40% of people with migraine do not respond sufficiently to triptan therapy in controlled trials [21–24].

In the current study, levels of satisfaction with acute therapy were lower in insufficient responders than in sufficient responders, with insufficient responders being significantly less likely than sufficient responders to express a willingness to continue with their current prescribed acute therapy. Overall, therefore, this analysis of Japan Adelphi Migraine DSP data supports previous findings of high levels of unmet need due to an insufficient response to current acute treatment options for people with migraine [5, 25].

Further real-world evidence that people with migraine in Japan have unmet treatment needs, albeit with respect to preventive medications, was also seen in a 2019 analysis of the Japan Medical Data Center claims database. This study reported that only 15% of people identified with migraine had received preventive medication as their index migraine treatment regimen and that, among these people, discontinuation of initial preventive treatment was common (67–83%), occurred following only a short period of treatment, and was ongoing, with most (85% of discontinuers) continuing to receive no preventive treatment after discontinuation [26].

Factors associated with insufficient response to prescribed acute treatment identified in the logistic model of this analysis of real-world data from Japan included taking acute prescribed medication when/after pain started versus at first sign of migraine, seeing a neurologist rather than an internist, having cardiovascular disease, and a higher MIDAS score. Few previous studies have investigated factors associated with response to acute therapy in people with migraine, but similar findings were reported in an analysis of the 2014 US Adelphi Migraine Specific DSP data [19]. In that analysis, taking acute medication when/after pain started (vs. at first sign of migraine) and higher MIDAS total scores were also associated with insufficient response to acute treatment. The study additionally identified depression as significantly associated with insufficient response [19], a finding also reported by the US AMPP population-based study [25].

The current analysis of the Japan Adelphi Migraine DSP data found the presence of cardiovascular disease to be associated with a lower odds of an insufficient response to acute therapy. Cardiovascular disease (myocardial infarction, peripheral vascular disease, ischemic heart disease, stroke, transient ischemic attack and uncontrolled hypertension) is considered a contraindication to the use of triptans [27, 28], a widely used acute treatment for migraine [29]. In the Japan Adelphi Migraine DSP, a majority of acute prescriptions were for triptans and NSAIDs [8]; hence, this inverse association between cardiovascular disease and insufficient response to acute medication requires further study.

Other factors associated with an insufficient response to acute migraine treatment identified from clinical or population-based studies include older age, higher body mass index, greater headache severity, more headache days per month, presence of migraine-related symptoms (e.g., photophobia/phonophobia, nausea) or cutaneous allodynia, use of NSAIDs, and non-use of preventive migraine medications or triptans [6, 25, 30–34]. Notably, high levels of discontinuation of migraine therapies (triptans and preventive therapies), assessed using pharmacy claims data, were recently also found to be indicative of an insufficient patient response to acute treatment [35]. Conflicting findings have been reported for the influence of gender and the time of treatment administration on response to therapy [31–33].

The finding that early administration of acute treatment (at the first sign of a migraine attack rather than when/after the pain starts) reduced the risk of an insufficient response to such therapy has notable implications for people with migraine in Japan. Currently in Japan, prescriptions of acute medications, such as triptans, are limited to 10–14 doses/month, and routine use of opioids for treating migraine is off-label. Hence, some people with migraine will hesitate before taking acute therapy, postponing administration until the signs of a migraine attack are more pronounced, thus potentially increasing their chances of an insufficient response.

Consulting with a headache specialist rather than a non-specialist could increase a patient’s awareness of how to improve the management of their migraine attacks. For example, the patient could be encouraged to record their migraine experience (e.g., in a migraine headache diary), which could help them better control the timing of their acute medication administration. We found that seeing a neurologist rather than an internist was associated with a higher risk of insufficient response to acute treatment, which suggests both that insufficient responders may seek out the care of a headache specialist and that even people who are consulting headache specialists are struggling to adequately treat their migraine attacks.

The finding of an association between a higher MIDAS score and an insufficient response to prescribed acute treatment in the logistic model of this analysis suggests that patients with greater levels of migraine-related disability are also struggling to adequately treat their migraine attacks.

The implications of these findings, which suggest that the earlier patients receive effective acute treatment for their migraine attacks the better the impact on longer-term prognosis, warrant further study. In particular, more research is needed to clarify, for example, the differences in factors related to insufficient response to acute therapy reported between real-world studies and those of population-based and clinical studies.

We found treatment patterns to be largely similar between insufficient responders and sufficient responders to prescribed acute treatment in Japan. However, insufficient responders to acute treatment were more likely than sufficient responders to exhibit greater migraine severity, as indicated by the higher proportions of insufficient responders with a clinical diagnosis of chronic migraine or medication-overuse or tension-type headache and the greater requirements of insufficient responders for extra doses of prescribed acute medication to relieve pain symptoms or symptoms of a migraine attack. Headache-related disability was also significantly greater and HRQoL significantly lower in Japanese insufficient responders to acute treatment than in sufficient responders, and similar findings have been reported in the overall 2017 analysis of Adelphi Migraine DSP data from USA, France, Germany, Italy, Spain, and the UK [20] and in the 2014 US Adelphi Migraine DSP [19]. Comparison of the EQ-5D utility score for insufficient responders to acute therapy in the current study (0.847) with reported Japanese norms (0.950–0.899 for age range > 20–29 to < 70 years [36]) suggests that HRQoL is markedly impaired in Japanese people with migraine with an insufficient response to acute therapy.

Comparison of the Japan and US Adelphi Migraine DSP insufficient responder data reveals notably lower mean MIDAS scores in the Japanese cohort (12.7 vs. 21.0, indicating less migraine-related disability) and more frequent reports of little or no disability (69.1% vs. 31.5%) [19]. One reason for these differences could be that the proportion of insufficient responders experiencing a low frequency (0–3) of migraine headache days per month was higher in the Japanese cohort than in the corresponding US cohort (59.9% vs. 53.7%) [19]. Additionally, these findings are possibly indicative of cultural differences between Japan and the USA.

A comparison of Japan and US Adelphi Migraine DSP WPAI scores [19] indicates that migraine impacted impairment at work, overall work impairment, and usual activity to a significantly greater extent in insufficient responders than in sufficient responders in both countries. However, in the 2014 US Adelphi Migraine DSP, migraine also significantly impacted work time missed due to migraine [19]. In the 2017 analysis of Adelphi Migraine DSP data from USA, France, Germany, Italy, Spain, and the UK, significantly greater impairments in work productivity and activity were seen in insufficient responders to triptan therapy than in sufficient responders [20].

### Strengths/limitations

A major strength of this study is the use of real-world data collected using a standardized methodology (as part of the Adelphi Migraine DSP [9]), thus facilitating the comparison of study findings with those from other countries. Strengths and limitations of the use of Japan Adelphi Migraine DSP data have previously been reported [8].

As all patients who participated in the Japan Adelphi Migraine DSP had a physician-confirmed diagnosis of migraine, the study findings can be considered representative of consulting patients with migraine in Japan. However, it should be noted that consulting physicians were selected on the basis of the volume of patients with migraine routinely seen and hence had high levels of experience in treating migraine attacks. These results may therefore not be generalizable to the wider population with migraine.

Another strength of the study was that the backward logistic model used to identify factors associated with insufficient response to prescribed acute treatment included not only variables previously reported as associated with treatment response (e.g., older age, female sex, greater headache severity, and presence of migraine-related symptoms) but also additional patient characteristics and treatment patterns (e.g., numbers of prescribed acute and preventive medications), MIDAS total score and other HRQoL data, and time of administration of acute treatment.

Response to acute treatment was patient reported. There is currently no standard definition for assessing response to acute treatment for migraine. However, the definition of response we used was based on a recognized efficacy endpoint in clinical trials assessing acute treatments for migraine (headache pain freedom within 2 h of acute treatment in at least four of five [80%] migraine attacks) and is one that is desirable to patients [14]. Other researchers have proposed cut-offs for response as a positive outcome (pain freedom at 2 h) in at least two of three (67%) treated attacks [37] or three of four attacks (75% [38]). Hence, we believe that the definition we used to assess response to acute treatment was appropriate.

Additional limitations of the study include that the data are cross-sectional in nature (hence, causality cannot be inferred) and that only a limited number of physicians and patients participated.

## Conclusions

This analysis of Japan Adelphi Migraine DSP data suggests that many people with migraine in Japan are struggling to adequately treat their migraine attacks with prescribed acute medication and exhibit high levels of unmet acute treatment needs. A need therefore exists for an expansion in acute therapeutic options for people with migraine, the optimization of treatment involving new and existing acute therapies (with different dosages and with differing routes of administration), and preventive and interventional treatment approaches.

## Supplementary information

**Additional file 1 : Table S1.** Current migraine-related symptom severity by response to acute treatment for migraine.

**Additional file 2 : Figure S1.** Change in level of impairment over past 6 months by response to acute treatment for migraine.

## Data Availability

The data that support the findings of this study are available from Adelphi Real World, but restrictions apply to the availability of these data, which were used under license for the current study and so are not publicly available. However, data are available from the authors upon reasonable request and with permission from Adelphi Real World.

## Abbreviations

AMPP: American Migraine Prevalence and Prevention
CI: Confidence interval
COPD: Chronic obstructive pulmonary disease
DSP: Disease-Specific Programme
EQ-5D: EuroQol-5 Dimensions
GI: Gastrointestinal
HRQoL: Health-related quality of life
IHS: International Headache Society
JHS: Japanese Headache Society
JSN: Japanese Society of Neurology
MIDAS: Migraine disability assessment
NSAID: Nonsteroidal anti-inflammatory drug
OR: Odds ratio
OTC: Over the counter
PRF: Patient record form
PSC: Patient self-completion
SD: Standard deviation
WPAI: Work productivity and activity impairment
YLD: Years lived with disability
