# Supplementary material for: Factors associated with insufficient response to acute treatment of migraine in Japan: analysis of real-world data from the Adelphi Migraine Disease Specific Programme
Source: BMC Neurol. 2020 Jul 8;20:274. doi: 10.1186/s12883-020-01848-4 (PMC7341635; doi:10.1186/s12883-020-01848-4)
Supplement: Supplementary file 1 — Additional file 1 : Table S1. Current migraine-related symptom severity by response to acute treatment for migraine. [file 12883_2020_1848_MOESM1_ESM.docx]

**Supplementary Table 1.** Current migraine-related symptom severity by response to acute treatment for migraine

|  | **Insufficient responder** | | | | **Sufficient responder** | | | |  |
| --- | --- | --- | --- | --- | --- | --- | --- | --- | --- |
|  | **None** | **Mild** | **Moderate** | **Severe** | **None** | **Mild** | **Moderate** | **Severe** | **p-value** |
| Unilateral pain, n (%) * | 51 (22.5) | 24 (10.6) | 126 (55.5) | 26 (11.5) | 57 (18.3) | 63 (20.3) | 157 (50.5) | 34 (10.9) | 0.025 |
| Bilateral pain, n (%) * | 131 (57.7) | 14 (6.2) | 63 (27.8) | 19 (8.4) | 214 (68.8) | 22 (7.1) | 57 (18.3) | 18 (5.8) | 0.027 |
| Pulsating/throbbing pain, n (%) | 99 (43.6) | 19 (8.4) | 78 (34.4) | 31 (13.7) | 143 (46.0) | 41 (13.2) | 91 (29.3) | 36 (11.6) | 0.218 |
| Pain worsened by activity, n (%)* | 145 (63.9) | 19 (8.4) | 42 (18.5) | 21 (9.3) | 231 (74.3) | 15 (4.8) | 50 (16.1) | 15 (4.8) | 0.030 |
| Sensitivity to light (photophobia), n (%) | 136 (59.9) | 44 (19.4) | 34 (15.0) | 13 (5.7) | 213 (68.5) | 45 (14.5) | 43 (13.8) | 10 (3.2) | 0.141 |
| Sensitivity to sound (phonophobia), n (%) | 158 (69.6) | 29 (12.8) | 31 (13.7) | 9 (4.0) | 240 (77.2) | 32 (10.3) | 29 (9.3) | 10 (3.2) | 0.246 |
| Sensitivity to smell, n (%) * | 191 (84.1) | 15 (6.6) | 18 (7.9) | 3 (1.3) | 267 (85.9) | 24 (7.7) | 16 (5.1) | 4 (1.3) | 0.002 |
| Sensory aura, n (%) * | 194 (85.5) | 16 (7.0) | 15 (6.6) | 2 (0.9) | 290 (93.2) | 12 (3.9) | 6 (1.9) | 3 (1.0) | <0.001 |
| Nausea, n (%) | 96 (42.3) | 47 (20.7) | 65 (28.6) | 19 (8.4) | 165 (53.1) | 59 (19.0) | 72 (23.2) | 15 (4.8) | 0.058 |
| Vomiting, n (%) | 165 (72.7) | 22 (9.7) | 31 (13.7) | 9 (4.0) | 242 (77.8) | 29 (9.3) | 27 (8.7) | 13 (4.2) | 0.321 |
| Visual aura, n (%) * | 190 (83.7) | 16 (7.0) | 19 (8.4) | 2 (0.9) | 285 (91.6) | 16 (5.1) | 8 (2.6) | 2 (0.6) | <0.001 |
| Speech disturbance, n (%) * | 209 (92.1) | 16 (7.0) | 2 (0.9) | 0 | 306 (98.4) | 5 (1.6) | 0 | 0 | <0.001 |
| Muscle weakness/fatigue, n (%) * | 197 (86.8) | 18 (7.9) | 12 (5.3) | 0 | 294 (94.5) | 11 (3.5) | 6 (1.9) | 0 | 0.007 |
| Light-headedness, n (%) * | 201 (88.5) | 22 (9.7) | 4 (1.8) | 0 | 292 (93.9) | 12 (3.9) | 5 (1.6) | 2 (0.6) | <0.001 |

Physician-reported data

*p<0.05 between insufficient responders and sufficient responders across the range of severity using chi-squared or Fisher’s exact test. The four levels of severity are none, mild, moderate, and severe, which together make up 100%
