# Supplementary material for: Factors associated with insufficient response to acute treatment of migraine in Japan: analysis of real-world data from the Adelphi Migraine Disease Specific Programme
Source: BMC Neurol. 2020 Jul 8;20:274. doi: 10.1186/s12883-020-01848-4 (PMC7341635; doi:10.1186/s12883-020-01848-4)
Supplement: Supplementary file 2 — Additional file 2 : Figure S1. Change in level of impairment over past 6 months by response to acute treatment for migraine. [file 12883_2020_1848_MOESM2_ESM.docx]

**Supplementary Fig. 1** Change in level of impairment over past 6 months by response to acute treatment for migraine.


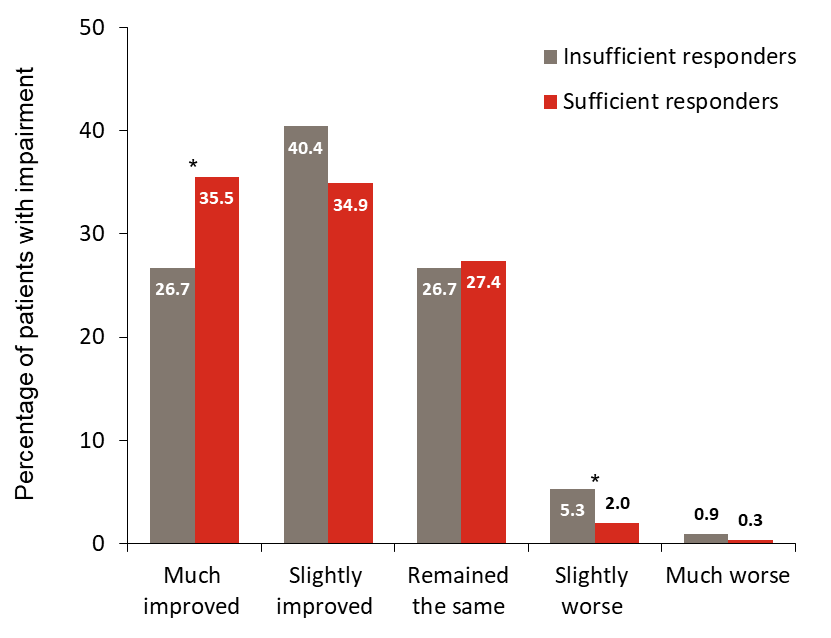


Physician-reported data. *p<0.05 between insufficient responders and sufficient responders using chi-squared or Fisher’s exact test. Percentages are calculated as proportion of non-missing data
